# Posterior keratometry changes after steep axis phacoemulsification: a prospective study

**DOI:** 10.22336/rjo.2023.45

**Published:** 2023

**Authors:** Jaya Kaushik, Ankita Singh, Sunandan Bhatta, Sumit Goyal, Jitendra Kumar Singh Parihar

**Affiliations:** * Department of Ophthalmology, Command Hospital (Lucknow), U.P, India; ** Department of Ophthalmology, Military Hospital (Bathinda), Punjab, India; *** Department of Ophthalmology, Military Hospital (Agra), U.P, India; **** Department of Ophthalmology, Military Hospital (Bareilly), U.P, India; ***** Post Graduate Training & Education, Centre for Sight, New Delhi, India

**Keywords:** keratometry, steep axis, phacoemulsification, astigmatism, Scheimpflug imaging

## Abstract

**Purpose:** To measure changes in posterior corneal curvature after steep axis phacoemulsification and investigate the possibility of its effect on SIA.

**Methods:** This was a prospective longitudinal study on 60 consecutive eyes of age-related cataract with regular astigmatism and absence of co-morbidities undergoing uneventful cataract surgeries with main incision at steep meridian. Preoperative and 4 weeks postoperative measurements of anterior and posterior corneal curvatures using Scheimpflug based corneal topographer were performed. Posterior corneal curvature was assessed at 3, 5- and 7-mm diameters.

**Results:** The study found a statistically significant change in posterior corneal K1, K2 and mean astigmatism in all zones (3, 5 and 7 mm) at 4 weeks postoperative, when compared to preoperative readings.

**Conclusion:** Posterior keratometry is likely to be an important determinant of Surgically Induced Astigmatism (SIA) and should be factored in for refractive cataract surgery.

## Introduction

Expectation of patients from cataract surgery with respect to visual rehabilitation is currently comparable to refractive surgery. In this regard, beyond the mandatory precision calculation with regards to lens power and position, corneal astigmatic changes after surgery have evolved as a major criterion affecting both visual acuity and quality of vision. Hence, intraoperative correction of corneal astigmatism is a mandatory requirement in the current scenario. It has become significantly important for the cataract surgeons to know the effect of their incisions on corneal astigmatism. 

The difference between the curvature of the steepest and flattest corneal meridians in diopter can give the measurement of regular corneal astigmatism. The refractive cataract surgery aims at decreasing the corneal astigmatism. Studies have shown that more than 30% of eyes in the normal population have a diopter (D) greater than 1.0 of regular corneal astigmatism [**1**,**2**]. Hence, there is an inflated demand to minimize corneal astigmatism during cataract surgery to obtain an optimum uncorrected visual acuity.

In the past, measurements of corneal astigmatism were limited to keratometry of central 3 mm of anterior cornea. At present, it is no longer sufficient. Moreover, in recent years, the influence of posterior corneal astigmatism on total corneal astigmatism has caused great concern in the clinical practice [**3**,**4**].

With the advent of the Scheimpflug anterior segment imaging system, the measurement of posterior corneal curvature has become a routine before planning the refractive cataract surgery [**5**-**9**].

In general, anterior corneal astigmatism is commonly measured neglecting the role of posterior corneal astigmatism. Previous studies have shown that “surgically induced astigmatism” (SIA) is calculated based on the anterior keratometric data without taking into consideration the effect of the incision on the posterior corneal curvature, which may lead to error in calculation of surgically induced astigmatism [**10**-**13**].

The aim of the present study was to analyze the effect of steep axis phaco emulsification on the posterior corneal curvature based on the keratometry readings derived from the rotating Scheimpflug imaging device.

## Methods

This prospective study was performed in accordance with the tenets of the Declaration of Helsinki and was approved by the Institutional Ethics Committee. An informed written consent was taken from all the patients after explaining the procedure involved in the study. There was no deviation from the standard of care in the treatment of patients enrolled in study.

This study included 60 eyes of consecutive patients with age-related cataract, who presented to the outpatient department and underwent phacoemulsification at this tertiary care eye center, from January 2023 to February 2023. The Lens Opacities Classification System III (LOCS III) was used to grade the cataract.

Inclusion criteria included:

• patients with age-related cataract;

• regular astigmatism by topography;

• no co-existing ocular pathology. 

Exclusion criteria included:

• cataract other than age-related;

• history of ocular trauma;

• contact lens wear;

• corneal scars; 

• opacities affecting visual outcomes.

All patients underwent a complete ophthalmic evaluation preoperatively that included visual acuity, slit lamp examination and optical biometry. Keratometry readings of anterior corneal surface and posterior corneal surface within radii of 3 mm, 5 mm and 7 mm respectively were obtained using a Scheimpflug tomographer (Schwind Sirius + corneal pachymetry and topography). Three images of the studied eye were captured with Sirius (Schwind Sirius + corneal pachymetry and topography). The device was set to a 25 im-ages/second mode and images were taken in auto mode. The software allowed for automatic analysis of the anterior segment, anterior and posterior topography, and keratometry of the cornea and anterior chamber anatomy. The parameters obtained were used to guide the position of the steep-axis Clear Corneal Incision according to the steepest corneal meridian. The anterior and posterior corneal astigmatism was defined as with-the-rule astigmatism, against-the-rule astigmatism and oblique, based on steep corneal curvature. Postoperatively, anterior and posterior corneal surface keratometric data and corneal topography data was obtained at 4 weeks following cataract surgery and compared with the preoperative data to analyze the difference in study variables. Also, postoperative refractive outcome was measured for all the patients in the form of residual sphere, cylinder and spherical equivalent.

**Surgical Procedure**

All eyes underwent phacoemulsification surgery with single piece foldable aspheric monofocal posterior chamber intraocular lens. The surgery was performed by the same surgeon under topical anesthesia. A 2.8 mm biplanar corneal incision was created at the steep meridian of true calculated corneal power with a 2.8 mm double bevel disposable knife (micro-ophthalmic knife M2C K 2.8 mm). Two 1mm side ports were created at 90o on either side of the 2.8 mm incision using a 15o side port disposable blade (micro-ophthalmic knife M1K 15 deg). A continuous curvilinear capsulorhexis (CCCC) was performed with a cystotome, after which hydrodissection and hydrodelineation was done, and nuclear emulsification was performed using the Direct phaco chop technique. All surgeries were performed using the Alcon Infiniti® phacoemulsification system (Alcon, America) using torsional phaco in burst mode. The ultrasound and fluidic settings were as follows: bottle height, 95 cm; OZil amplitude 80% (torsional) linear pedal control; vacuum 400 mm Hg; and aspiration flow, 35 mL/min fixed pedal control. A foldable monofocal IOL (AMO Tecnis 1) was implanted in the capsular bag without enlarging the main incision. At the end of the surgery, the corneal incisions were hydrated. Postoperatively, patients received a combination of moxifloxacin and dexamethasone in tapering doses. All eyes were evaluated with the Scheimpflug tomographer for postoperative analysis after 4 weeks of cataract surgery.

**Statistical Analysis**

Statistical analysis was performed using IBM SPSS v25 (Statistical Product and Service Solutions) software. Descriptive statistical results were described as mean, standard deviation, range and 95% confidence interval (95% CI) from the mean. Measurable values like age, sex and laterality of eyes were considered categorical variables and were described as percentages. Parametric tests like paired t-test and Pearson’s Correlation were used to statistically analyze normally distributed data. Paired sample t-test was used to analyze the change in anterior K1 and K2 at the two time points. Non-parametric tests like Wilcoxon signed-rank test and Spearman Correlation were used to statistically analyze data that was not normally distributed. P value < 0.05 was considered to be significant. 

## Results

A total of 60 patients participated in this study. One eye of each patient was considered. The mean age (years) of the patients was 61.48 ± 8.97. 31 (51.7%) participants were males, while the remaining 29 (48.3%) participants were females. 33 (55.0%) patients underwent surgery of right eye (OD) and 27 (45.0%) patients underwent surgery of left eye (OS). 39 (65.0%) patients had pre-existing astigmatism “against the rule” (ATR), while 15 (25.0%) patients had astigmatism “with the rule” (WTR) and the remaining 6 (10.0%) patients had oblique astigmatism. The association between the types of astigmatism and the other parameters is mentioned in **Table 1**.

**Table 1:** Association between Type of Astigmatism and Parameters

	Type of Astigmatism			
Parameters	ATR (n = 39)	WTR (n = 15)	Oblique (n = 6)	p value
Axial Length	23.32 ± 0.82	23.54 ± 1.01	22.50 ± 0.82	0.083 1
ACD (mm)	3.21 ± 0.40	3.40 ± 0.39	2.95 ± 0.46	0.091 1
SE (Postoperative)	-0.30 ± 0.68	-0.73 ± 0.71	-0.56 ± 0.50	0.111 1
Anterior K1 (Preoperative)	43.52 ± 1.39	43.22 ± 2.04	44.71 ± 1.41	0.180 1
Anterior K2 (Preoperative)	44.29 ± 1.27	43.99 ± 2.10	45.27 ± 1.30	0.204 1
Cylinder (Anterior) (D) (Preoperative)	0.77 ± 0.40	0.77 ± 0.44	0.67 ± 0.29	0.850 1
Posterior K1 (3 mm) (Preoperative)	-6.18 ± 0.19	-6.12 ± 0.34	-6.24 ± 0.25	0.509 1
Posterior K2 (3 mm) (Preoperative)	-6.41 ± 0.25	-6.46 ± 0.37	-6.54 ± 0.15	0.491 1
Cylinder (Posterior) (3 mm) (D) (Preoperative)	0.23 ± 0.12	0.34 ± 0.17	0.30 ± 0.16	0.111 1
Posterior K1 (5 mm) (Preoperative)	-6.16 ± 0.18	-6.10 ± 0.33	-6.21 ± 0.27	0.435 1
Posterior K2 (5 mm) (Preoperative)	-6.36 ± 0.23	-6.40 ± 0.36	-6.50 ± 0.17	0.463 1
Cylinder (Posterior) (5 mm) (D) (Preoperative)	0.20 ± 0.10	0.29 ± 0.16	0.21 ± 0.06	0.104 1
Posterior K1 (7 mm) (Preoperative)	-6.03 ± 0.49	-6.07 ± 0.34	-6.21 ± 0.23	0.409 1
Posterior K2 (7 mm) (Preoperative)	-6.30 ± 0.21	-6.29 ± 0.42	-6.40 ± 0.23	0.674 1
Cylinder (Posterior) (7 mm) (D) (Preoperative)	0.26 ± 0.46	0.26 ± 0.15	0.18 ± 0.08	0.326 1
Anterior K1 (Postoperative)	43.64 ± 1.54	43.37 ± 2.09	44.74 ± 1.06	0.201 1
Anterior K2 (Postoperative)	44.35 ± 1.48	44.26 ± 1.87	45.42 ± 1.09	0.186 1
Cylinder (Anterior) (D) (Postoperative)	-0.65 ± 0.46	-0.68 ± 0.55	-0.68 ± 0.31	0.766 1
Cylinder Axis (Anterior) (Postoperative)***	71.28 ± 38.17	112.33 ± 51.93	87.33 ± 39.45	0.020 1
Posterior K1 (3 mm) (Postoperative)	-6.23 ± 0.22	-6.20 ± 0.38	-6.34 ± 0.11	0.559 1
Posterior K2 (3 mm) (Postoperative)	-6.55 ± 0.26	-6.75 ± 0.50	-6.69 ± 0.26	0.190 1
Cylinder (Posterior) (3 mm) (D) (Postoperative)	0.30 ± 0.16	0.58 ± 0.50	0.31 ± 0.19	0.052 1
Posterior K1 (5 mm) (Postoperative)	-6.24 ± 0.17	-6.20 ± 0.35	-6.35 ± 0.16	0.398 1
Posterior K2 (5 mm) (Postoperative)	-6.50 ± 0.27	-6.71 ± 0.41	-6.62 ± 0.21	0.096 1
Cylinder (Posterior) (5 mm) (D) (Postoperative)***	0.26 ± 0.17	0.51 ± 0.39	0.28 ± 0.13	0.013 1
Posterior K1 (7 mm) (Postoperative)	-6.20 ± 0.17	-6.24 ± 0.36	-6.29 ± 0.22	0.695 1
Posterior K2 (7 mm) (Postoperative)	-6.42 ± 0.24	-6.57 ± 0.32	-6.53 ± 0.18	0.119 1
Cylinder (Posterior) (7 mm) (D) (Postoperative)***	0.22 ± 0.12	0.40 ± 0.22	0.24 ± 0.15	0.008 1
***Significant at p<0.05, 1: Kruskal Wallis Test				

The mean axial length (mm) was 23.29 ± 0.90. The mean anterior chamber depth (mm) was 3.23 ± 0.42. The average mean pupillary power (MPP) in diopters (D) was 43.40 ± 1.48, while the average Cylinder MPP (D) was -1.02 ± 0.50 and the mean of the MPP Cylinder Axis (in degrees) was 95.35 ± 43.06. The mean postoperative spherical equivalent was -0.44 ± 0.69. The mean preoperative anterior corneal K1 was 43.56 ± 1.60 and K2 was 44.31 ± 1.53. The mean preoperative anterior corneal astigmatism (D) was 0.76 ± 0.40 with the mean axis (in degrees) of 91.68 ± 64.24. The mean **preoperative posterior keratometry values** within 3 mm were: K1 -6.17 ± 0.24, K2 -6.44 ± 0.28, mean astigmatism (D) 0.27 ± 0.15; within 5 mm radius were: K1 -6.15 ± 0.23, K2 -6.38 ± 0.26, mean astigmatism (D) 0.22 ± 0.12; within 7 mm radius were: K1 -6.06 ± 0.44, K2 -6.31 ± 0.28, mean astigmatism (D) 0.25 ± 0.38.

The mean postoperative anterior corneal K1 was 43.68 ± 1.67, K2 was 44.44 ± 1.57, anterior corneal astigmatism (D) was -0.66 ± 0.47, with the mean axis (in degrees) as 83.15 ± 44.96. The **mean postoperative posterior keratometry values** within 3 mm were: K1 -6.24 ± 0.26, K2 -6.61 ± 0.34, mean astigmatism (D) 0.37 ± 0.31; within 5 mm radius were K1 -6.24 ± 0.22, K2 -6.57 ± 0.31, mean astigmatism (D) 0.33 ± 0.26; within 7 mm radius were K1 -6.22 ± 0.23, K2 -6.47 ± 0.26, mean astigmatism (D) 0.27 ± 0.17.

**Graph 1:**
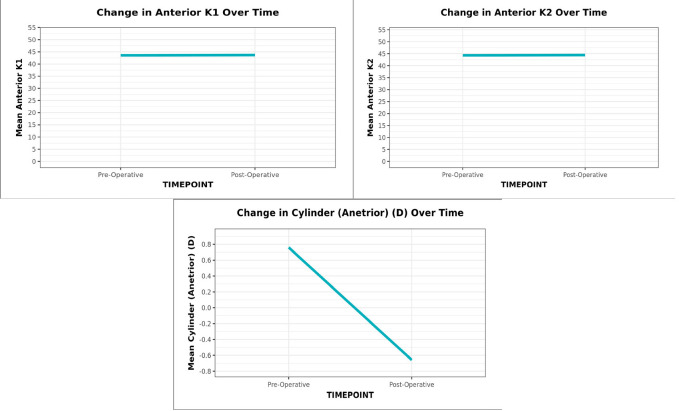
The line diagram depicts the change in Anterior K1, K2 and cylinder over time

The mean change in anterior K1 was 0.12 ± 0.85 D (Paired t-test: t = -1.1, p = 0.276); from a minimum value of 43.68 D preoperatively to a maximum value of 43.56 D postoperatively and anterior K2 was 0.13 ± 0.91 (Paired t-test: t = -1.1, p = 0.292); from a minimum value of 44.31 D preoperatively to maximum value of 44.44 D postoperatively, which was not statistically significant. However, the mean cylinder of anterior astigmatism (D) decreased from 0.76 ± 0.40 preoperatively to -0.66 ± 0.47 postoperatively, which was statistically significant (Wilcoxon Test, p = <0.001) (**Graph 1**). 

**Graph 2:**
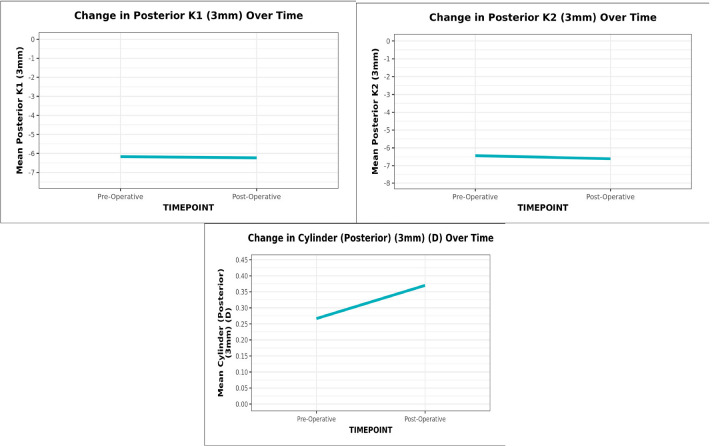
The line diagram depicts the change in Posterior K1, K2 and cylinder (3 mm) over time

The mean (SD) posterior K1 (3 mm) decreased from -6.17 (0.24) preoperatively to -6.24 (0.26) postoperatively, while posterior K2 (3 mm) decreased from -6.64 (0.28) to -6.61 (0.34) with an increase in astigmatism from 0.27 (0.15) to 0.37 (0.31), all three being statistically significant (Wilcoxon Test, p = <0.001) (**Graph 2**).

**Graph 3:**
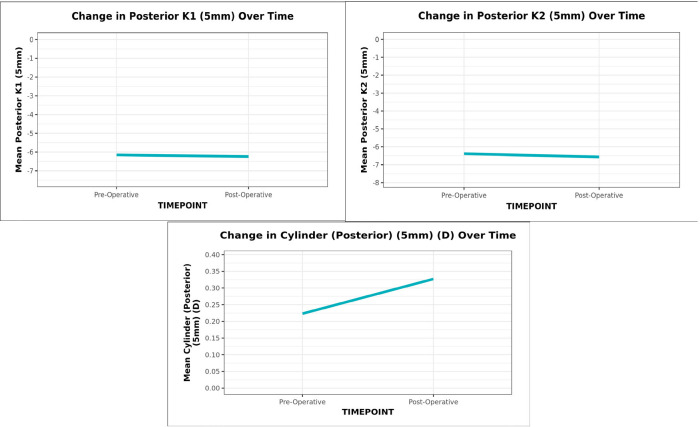
The line diagram depicts the change in Posterior K1, K2 and cylinder (5 mm) over time

The mean (SD) posterior K1 (5 mm) decreased from -6.15 (0.23) preoperatively to -6.24 (0.22) postoperatively, while posterior K2 (5 mm) decreased from -6.38 (0.26) to -6.57 (0.31) with an increase in astigmatism from 0.22 (0.12) to 0.33 (0.26), all three being statistically significant (Paired t-test, p = <0.001) (**Graph 3**).

**Graph 4:**
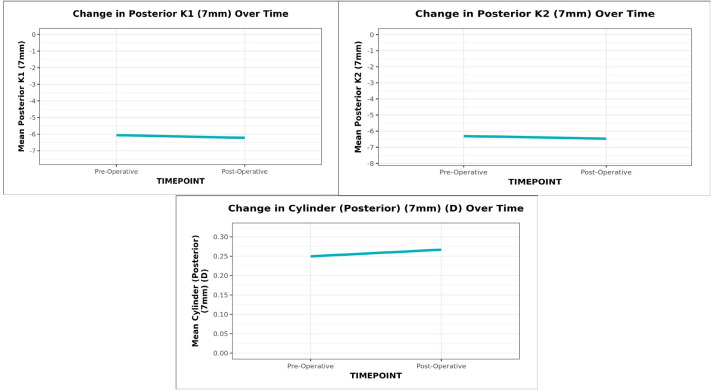
The line diagram depicts the change in Posterior K1, K2 and cylinder (7 mm) over time

The mean (SD) posterior K1 (7 mm) decreased from -6.06 (0.44) preoperatively to -6.22 (0.23) postoperatively and Posterior K2 (7 mm) decreased from -6.31 (0.28) to -6.47 (0.26) with an increase in astigmatism from 0.25 (0.38) to 0.27 (0.17), all three being statistically significant (Wilcoxon Test: p = <0.001) (**Graph 4**).

## Discussion

**Graph 5:**
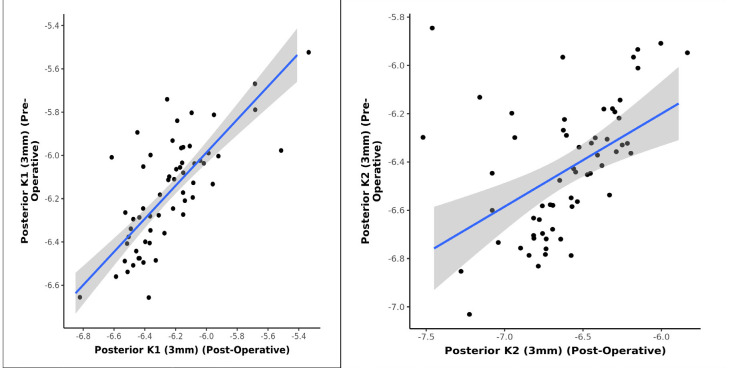
Correlation between Posterior K1, K2 (3 mm) - Preoperative and Posterior K1, K2 (3 mm) - Postoperative (n = 60)

**Graph 6:**
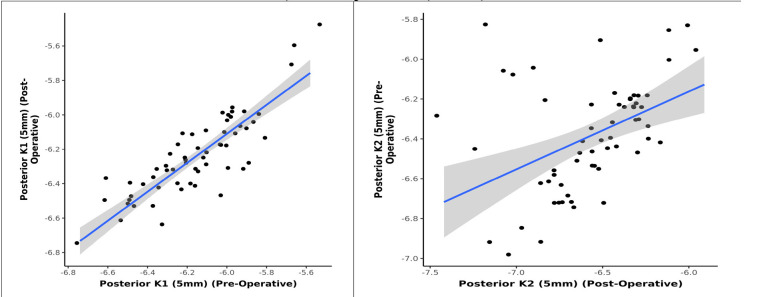
Correlation between Posterior K1, K2 (5 mm) - Preoperative and Posterior K1, K2 (5 mm) - Postoperative (n = 60)

**Graph 7:**
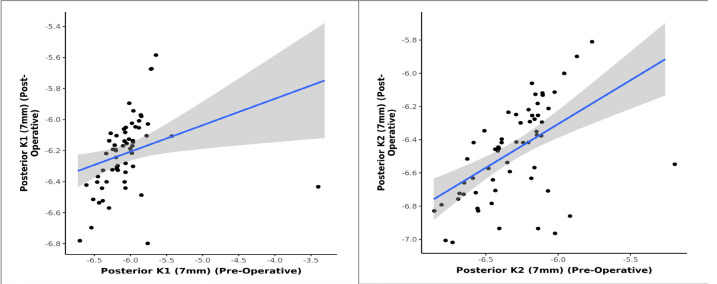
Correlation between Posterior K1, K2 (7 mm) - Preoperative and Posterior K1, K2 (7 mm) - Postoperative (n = 60)

The study investigated any significant change of keratometry parameters of posterior cornea following phacoemulsification cataract surgery. 60 eyes with pre-existing regular astigmatism were analyzed, which underwent steep axis phacoemulsification to observe any change on the posterior corneal curvature. No statistically significant change in the anterior corneal keratometry (K1, K2) was observed. However, there was a statistically significant change in the anterior corneal astigmatism. A statistically significant change was also observed in posterior corneal keratometric values and astigmatism in the postoperative follow-up period. A strong positive correlation between pre and postoperative posterior K1 and K2 (3 mm, 5 mm, 7 mm) was noted, and this correlation was statistically significant (Pearson’s Correlation, p = <0.001). The statistical analysis also showed that for every 1-unit increase in the preoperative value of posterior K1 (3 mm), the postoperative value increased by 0.87 units and for every 1-unit increase in the preoperative value of posterior K2 (3 mm), the postoperative value increased by 0.57 units (**Graph 5**). Similarly, for every 1-unit increase in K1 (5 mm) (preoperative), the postoperative posterior K1 (5 mm) increased by 0.84 units and for every 1-unit increase in the preoperative value of posterior K2 (5 mm), the postoperative value increased by 0.55 units (**Graph 6**). Similarly, for every 1-unit increase in K1 (7 mm) (preoperative), the posterior K1 (7 mm) (preoperative) increased by 0.80 units and for every 1-unit increase in the preoperative value of posterior K2 (7 mm), the postoperative value increased by 0.53 units (**Graph 7**).

The correction of the pre-existing corneal astigmatism is one of the major goals of the modern-day refractive cataract surgery. The conventional practice includes correcting anterior corneal astigmatism due to the lack of instruments that can accurately measure posterior or total corneal astigmatism. However, with the advent of the Scheimpflug anterior segment analysis system, the measurement of astigmatism on the posterior cornea can be achieved [**14**,**15**]. It has been observed that the posterior cornea acts as a minus lens, having astigmatism “with the rule” [**16**,**17**]. As age advances, the meridian of posterior corneal astigmatism remains stable, but the anterior corneal astigmatism changes from “with the rule” to “against the rule” [**18**,**19**]. Therefore, theoretically, we need to leave the corneas with slight astigmatism “with the rule” to compensate for this age-related change. Cheng et al. found a significant correlation between the magnitude of the Pentacam-derived posterior SIA and that of the anterior surface [**20**].

These studies have elicited that modern-day cataract surgery may alter the biomechanics of the cornea and this change can be related to the size of surgical incisions [**14**-**17**]. Corneal incisions can alter pre-existing corneal astigmatism. Until recently, no study has found significant correlation between posterior corneal astigmatism and surgical incision as so far, the precise measurement of the corneal posterior surface was not easily available.

The posterior corneal curvature may be clinically relevant for the correction of total astigmatism. The residual refractive errors after cataract surgery in normal eyes can probably be explained by the neglection of the effect of the posterior corneal curvature and its significant altered dynamics after cataract surgeries. Bregnhøj et al. suggested that the post-cataract surgery residual astigmatism largely represents posterior corneal astigmatism [**21**]. 

Koch et al. measured -0.3 D as the mean magnitude of posterior corneal astigmatism with a rotating dual Scheimpflug analyzer using ray-tracing that was greater than 0.50 D in 9.0% of cases [**22**].

Earlier results suggest that the neglection of the contribution of the posterior corneal surface may cause a significant error in estimating the total corneal SIA [**20**,**23**,**24**]. 

In the current study, there was a focal increase in posterior astigmatism following phacoemulsification at 4 weeks within 3 mm, 5 mm and 7 mm radii of curvature, which was statistically significant. In a study by Schmitt et al. of 2.75 mm corneal incision, in which they have studied posterior corneal curvature changes, similar focal steepening was seen both in K1 and K2. 

Thus, posterior SIA seems to have an individual distribution similar to that of anterior. Despite the novelty of this study that tried to evaluate the changes of posterior corneal curvature with steep axis phacoemulsification, the study was limited due to a relatively small study population (60 eyes) and the data from a single follow-up period (4 weeks). Further investigations with a larger sample population are imperative to establish a more comprehensive correlation. Also, a long-term follow-up can help to understand delayed changes in posterior corneal curvature. 

## Conclusion

Based on the results of our study, cataract surgery incision on the steep axis may have a larger impact on the posterior corneal surface than was previously thought. Posterior corneal astigmatism may have a significant clinical impact in the more precise planning of postoperative astigmatism after cataract surgeries. 

**Conflict of Interest**

The authors declare that there is no conflict of interest.

**Informed Consent and Human and Animal Rights statements**

Written informed consent has been obtained from the individuals involved in the study. 

**Authorization for the use of human subjects**

Ethical approval: The research related to human use complies with all the relevant national regulations, institutional policies, is in accordance with the tenets of the Helsinki Declaration, and has been approved by the ethics committee of Military Hospital (Bathinda), Punjab, India.

**Acknowledgements**

Nil.

**Sources of Funding**

The author(s) received no financial support for the research, authorship and/or publication of this article.

**Disclosures**

None.
